# The research output on interventions for the behavioural risk factors alcohol & drug use and dietary risk is not related to their respective burden of ill health in countries at differing World Bank income levels

**DOI:** 10.7189/jogh.10.020401

**Published:** 2020-12

**Authors:** Carlo Frassetto, Meisser Madera, Maximilian Siebert, Karen Megranahan, David Roberts, Emma Plugge

**Affiliations:** 1 School of Nursing, Catholic University of Sacred Hearth, Rome, Italy; 2 Department of Research, Faculty of Dentistry, University of Cartagena, Cartagena, Colombia; 3 Univ Rennes, CHU Rennes, Inserm, CIC 1414 (Centre d’Investigation Clinique de Rennes), Rennes, France; 4 Institute of Creative and Cultural Entrepreneurship, Goldsmiths College, University of London, London, UK; 5 Oxford School of Public Health, Nuffield Department of Population Health, University of Oxford, Oxford, UK; 6 Cochrane UK, Oxford, UK

## Abstract

**Background:**

Alcohol and drug use (A&D) and dietary risks are two increasingly important risk factors. This study examines whether there is a relationship between the burden of these risk factors in countries of specific income bands as defined by the World Bank, and the number of primary studies included in Cochrane Systematic Reviews (CSRs) conducted in those countries.

**Methods:**

Data was extracted from primary studies included in CSRs assessing two risk factors as outcomes. For each risk factor, data was obtained on its overall burden in disability-adjusted life years (DALYs) by World Bank Income Levels and examined for a link between DALYs, the number of primary studies and participants.

**Results:**

A total of 1601 studies from 95 CSRs were included. Only 18.3% of the global burden for A&D is in high income-countries (HICs) but they produced 90.5% of primary studies and include 99.5% of participants. Only 14.2% of the dietary risk burden is in HICs but they produced 80.5% of primary studies and included 98.1% of participants.

**Conclusions:**

This study demonstrates the unequal output of research heavily weighted towards HICs. More initiatives with informed contextual understanding are required to address this inequality and promote health research in low and middle-income countries.

Health research is important in informing improvements in health, and systematic reviews are key in developing the evidence base. However, research volume, in terms of Cochrane Systematic Review (CSR) output of reviews of interventions addressing specific risk factors, has been demonstrated by Roberts et al. to correlate only weakly with global risk burden [1,2]. In addition to this inequity of research against risk factor burden, they also noted an inequity in terms of where research occurred: only 19% of the corresponding authors in the CSRs studied were from low and middle income countries (LMICs) even though more than 83% of the global population live in these areas of the world [3] and incur most of the global risk factor burden.

To investigate the inequalities further, two behavioural risk factors were examined: alcohol and drug use (A&D) and dietary risks. These risk factors were selected because Roberts et al showed that there was a higher than expected number of reviews given the disease burden addressing the risk factor of A&D but a lower than expected number addressing dietary risks. The Global Burden of Disease (GBD) study measures the burden of risk factors in disability-adjusted life years (DALYs), that is, the sum of years lived with disability and years of life lost to premature death. The 2016 GBD study shows behavioural risk factors accounted for 32.7% (95% confidence interval 30.7-34.8) of attributable DALYs and highlights the importance of dietary risk factors and A&D risk factors in LMICs as well as in high income countries (HICs) [4].

The aim is to determine whether the burden of each of these two risk factors in countries of a particular income band (defined by the World Bank), and the number of primary studies and of study participants included in Cochrane Systematic Reviews conducted in those countries are related.

## METHODS

The Cochrane Database of Systematic Reviews via the Cochrane Library was searched using two filters (intervention and custom range date) to identify relevant systematic reviews published from 1^st^ January 2013 to the 2^nd^ October 2018. This five-year period was decided because it represents the median life expectancy of systematic reviews [5]. All primary intervention studies of any study design included in CSRs of interventions related to two modifiable behavioural risk factors (A&D and dietary risks) were selected for data extraction. When several versions of a published review were identified, the most recent publication was considered. Protocols and systematic reviews about diagnosis, prognosis, methodology and overviews were excluded.

All titles and abstracts were independently screened to exclude irrelevant reviews. Full texts were obtained for a final decision where necessary. Any disagreements were resolved by discussion, and an additional reviewer participated until an agreement was reached. Each included CSR was independently reviewed by two of the team of reviewing authors (CF, KM, MS, MM) to identify primary studies assessing interventions addressing diet and A&D.

The reviewing authors independently extracted data on the characteristics of primary studies including publication year, study design, country of first author, country where study took place, and number of participants. A 10% random selection of the extracted data was checked by one of the other reviewers. For each risk factor assessed, we obtained data on its overall burden in DALYs by World Bank Country Income Levels (WBIL) grouped into high, upper-middle, lower-middle and low income from the Global Burden of Disease study 2016 [6].

### Data analysis

A descriptive analysis of characteristics of the included primary studies was performed. Data was analysed and presented in tables using frequency and percentage for categorical variables. We used the Chi squared Goodness of Fit (CSGOF) to test the null hypothesis that the number of primary studies and the number of DALYs for that risk factor grouped by income band (high income vs upper middle, lower middle and low-income countries) were of the same distribution. This was repeated for the number of study participants. All statistical analyses were performed with STATA version 11 software [7] assuming a significance level of 0.05.

## RESULTS

**Figure 1:**
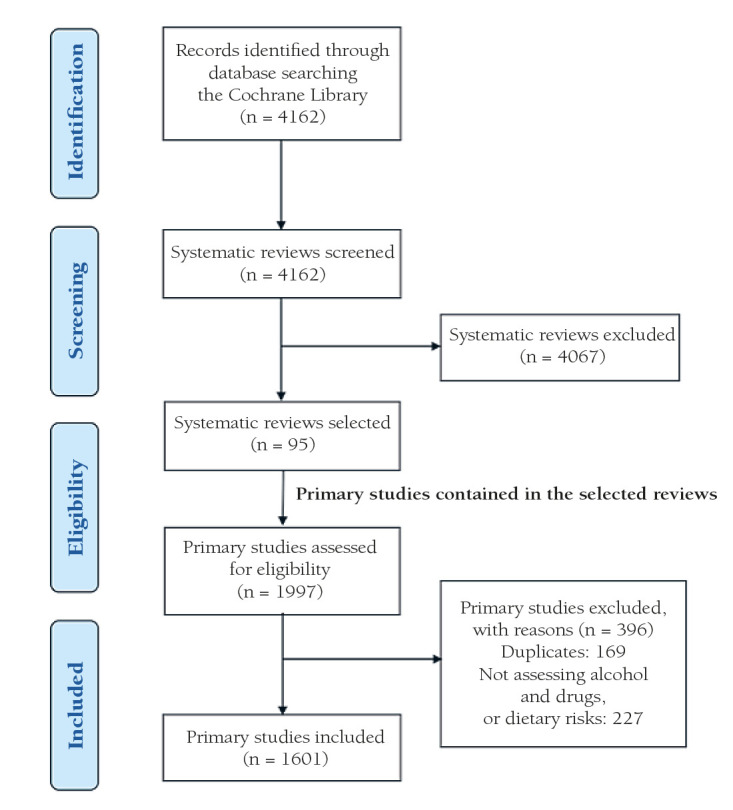
Prisma diagram of study selection.

A total of 95 CSRs met the inclusion criteria; 45 (47.4%) addressed dietary risks, 47 (49.5%) addressed A&D and three addressed (3.1%) both factors; and included 1601 primary studies. The selection process of the systematic reviews and its respective primary studies is shown in **Figure 1**. 95.6% of the primary studies were RCT’s; whereas the A&D section counted 97.1% RCTs and the dietary risks counted 94.3%. A total of 86.8% of the included studies were conducted in HICs; in A&D this made up a percentage 94.5% and in dietary risks 80.5%. For further characteristics of primary studies included in Cochrane systematic reviews by assessed risk factors please refer to **Table 1**.

**Table 1:** Characteristics of primary studies included in Cochrane systematic reviews by assessed risk factors

	Alcohol & drug use		Dietary risks	Total
**Publication year**	**n (%)**		**n (%)**	**n (%)**
2011-2018	194 (26.7)		369 (42.1)	563 (35.2)
2001-2010	356 (49.1)		320 (36.5)	676 (42.2)
1991-2000	131 (18.1)		142 (16.2)	273 (17.0)
≤1990	44 (6.1)		45 (5.1)	89 (5.6)
**Study design:**				
Cross-sectional	0 (0)		5 (0.6)	5 (0.3)
Cohort	9 (1.3)		38 (4.3)	47 (3.0)
NRS	1 (0.1)		6 (0.7)	7 (0.4)
Quasi-RCT	11 (1.5)		1 (0.1)	12 (0.7)
RCT	704 (97.1)		826 (94.3)	1530 (95.6)
**Country (World Bank income level):**				
High		685 (94.5)	705 (80.5)	1390 (86.8)
Upper middle		33 (4.5)	89 (10.2)	122 (7.6)
Lower middle		7 (1.0)	59 (6.7)	66 (4.1)
Low		0 (0)	23 (2.6)	23 (1.5)
**TOTAL**		725 (100)	876 (100)	1601 (100)

NRS - no randomized controlled study, RCT - randomised controlled trial

The majority of the burden for A&D was in LMICs but they only produced 5.5% of primary studies and 0.5% of participants were from these countries. Likewise, LMICs carried 85.8% of the burden for dietary risks but produced only 19.5% of primary studies and included 1.9% of participants. Details of the characteristics for the assessed risk factors by country income level can be found in **Table 2**.

**Table 2:** Characteristics for the assessed risk factors by country income level

Country income level	DALYs, n in millions (%)	Primary studies, n (%)	Country of primary study 1st author, n (%)	Participants primary studies, n (%)
**Alcohol & drugs use:**				
High	24.8 (18.3)	685 (94.5)	689 (95)	469 607 (99.5)
Upper middle	57.9 (42.7)	33 (4.5)	30 (4.1)	1929 (0.4)
Lower middle	39.2 (28.9)	7 (1.0)	6 (0.9)	472 (0.1)
Low	13.6 (10)	0 (0)	0 (0)	0 (0)
**TOTAL**	135.5 (100)	725 (100)	725 (100)	472 008 (100)
**Dietary risks:**				
High	32.4 (14.2)	705 (80.5)	738 (84.2)	530 009 (98.1)
Upper middle	91.8 (40.2)	89 (10.2)	87 (10.0)	6186 (1.1)
Lower middle	92.2 (40.3)	59 (6.7)	44 (5.0)	3050 (0.6)
Low	12.2 (5.3)	23 (2.6)	7 (0.8)	1256 (0.2)
**TOTAL**	228.6 (100)	876 (100)	876 (100)	540 501 (100)

DALYs – disability-adjusted life years

The χ^2^ goodness of fit (CSGOF) test showed the number of primary studies was of a different distribution to both risk factors studied; A&D risk factor burden in HICs and LMICs (*P* < 0.001), and dietary risk factor burden (*P* < 0.001). The number of study participants was also of a different distribution to the A&D risk factor burden in HICs and LMICs (*P* < 0.001) and also between dietary risk factor burden (*P* < 0.001).

## DISCUSSION

The authors examined 1601 primary studies from 95 CSRs. The results show 85.8% of the burden of disease for dietary risks is borne by LMICs yet only 19.5% of the primary studies and 1.9% of participants came from LMICs. The pattern is even starker for A&D since 81.7% of the burden of disease related to A&D is borne by LMICs but only 5.5% of the primary studies and 0.5% of participants came from LMICs. Notably 1390 (86.8%) of the 1601 primary studies examined were conducted in HICs. The total number of included studies taking place in the USA alone was 836, two thirds of those in HICs.

This study shows that the output of primary studies for the two behavioural risk factors, diet and A&D, are not in line with their respective burden of ill health in countries at differing WBIL.

The findings were similar to the ones of other studies conducted around these two risk factors. A previous study had shown that there were a higher number of CSRs addressing A&D than expected given risk factor disease burden [2], however, this study has identified that there is in fact a need for further research addressing A&D specifically in LMICs despite the fact that the risk factor of A&D was ‘over studied’ at a global level.

Also shown is the globally ‘understudied’ risk factor of poor diet is severely ‘understudied’ in LMICs. The World Health Organisation’s 2012 Strategy on Research for Health recognised the need to address “gaps in national and global research on health and health systems” [1]; this study has highlighted specific gaps and also the marked inequity in research outputs between HICs and LMICs.

Dietary risk is an increasing health issue for many countries [8] and is evident in countries of all income levels but for different reasons. In LMICs the reasons are particularly complex as populations in these countries experience under and over nutrition. Factors such as fast-food outlets, associated with poor diet and obesity in HICs, are increasingly an issue in LMICs, alongside poor sanitation or lack of food [8,9]. This highlights the urgent need for context specific research on effective interventions in these countries.

There is no doubt that A&D is on the increase [10,11]. Alcohol was reported as the 7^th^ highest risk factor for deaths in 2016 [12] and drug related overdose deaths in the USA alone had increased by 10% per year since 1999 [13]. Again, this highlights the urgent need to develop the evidence base for effective interventions addressing A&D in LMICs.

To the authors knowledge this is the first study that examines the burden of two behavioural risk factors, diet and A&D, in countries of specific World Bank income bands and how it relates to the number of primary studies and their participants included in CSRs conducted in those countries. The study was rigorously conducted, with two reviewers looking at a sample of all data with 92.6% agreement.

Limitations of the study were that only primary studies eligible for inclusion in CSRs were considered and other systematic reviews could have been included. The sources of bias in the included systematic reviews was not investigated. Furthermore because of resource constraints, the reviewing authors were only able to examine two specific risk factors, dietary risk and A&D, rather than all GBD level 2 modifiable risk factors. Finally, the identification of factors related to the burden of ill health is only exploratory. Several unmeasured confounders, for instance the difference in rural and urban areas, the cultural settings or the different quality of studies, could account for some of the findings. Other unmeasured confounders may exist, and great caution is warranted in interpreting these results, which naturally cannot be considered as reflecting any causal relationship.

The results of this research highlight that more primary research needs to be conducted in LMICs for the two behavioural risk factors studied; it should not be assumed that what is effective in HICs will also be effective in LMICs, although some interventions may indeed be globally relevant. This is part of a much wider problem regarding inequities in global health research. Factors hindering research equity in LMICs are worthy of further investigation such as sources of funding, language barriers to publication and broader socio-economic issues. HICs can support justice in global health by the design of grant programmes that will help adjust the disparity [14]. Clearly initiatives such as increased collaboration, capacity building initiatives for health research in LMICs, increased access to related literature and more funding opportunities for LMICs will help to start to address this imbalance [15].

In conclusion, this study demonstrates the inequal output of research heavily weighted towards HICs for A&D and Dietary risks. Therefore, more initiatives with informed contextual understanding are required to address this inequality and promote health research in low and middle-income countries.
